# Molecular mechanisms of peripheral nerve regeneration: emerging roles of microRNAs

**DOI:** 10.3389/fphys.2013.00055

**Published:** 2013-04-01

**Authors:** Di Wu, Alexander K. Murashov

**Affiliations:** ^1^Department of Physiology, East Carolina UniversityGreenville, NC, USA; ^2^Department of Neurobiology and Anatomy, Drexel University College of MedicinePhiladelphia, PA, USA

**Keywords:** miRNA, RNAi, nerve injury, nerve regeneration, translational regulation

## Abstract

MicroRNAs are small non-coding RNAs that suppress gene expression through target mRNA degradation or translation repression. Recent studies suggest that miRNA plays an important role in multiple physiological and pathological processes in the nervous system. In this review article, we described what is currently known about the mechanisms in peripheral nerve regeneration on cellular and molecular levels. Recently, changes in microRNA expression profiles have been detected in different injury models, and emerging evidence strongly indicates that these changes promote neurons to survive by shifting their physiology from maintaining structure and supporting synaptic transmission towards a regenerative phenotype. We reviewed the putative mechanisms involved in miRNA mediated post-transcriptional regulation and pointed out several areas where future research is necessary to advance our understanding of how targeting miRNA machinery can be used as a therapeutic approach for treating nerve injuries.

## Obstacles on the way of peripheral nerve regeneration

Nerve injuries induce severe disability and greatly compromise the quality of life. Injuries to peripheral nerves, occurring in approximately 2.8% of trauma patients, cause partial or total loss of motor, sensory and autonomic functions (Noble et al., 1998). Peripheral nerve injury leads to axon discontinuity and degeneration of myelinated fibers, which may eventually result in death of axotomized neurons (Navarro et al., 2007; Schuning et al., 2009). After peripheral nerve injury, the severed axons have the capability to regenerate and recover functional connections. However, a number of clinical reports and experimental studies in recent years also indicate that the rate of axonal regeneration is far from satisfactory, especially after severe injuries (Navarro et al., 2007).

The first detailed description on the repair of transected nerve trunks was recorded by Gabriele Ferrara in the sixteenth century (Ferrara, 1596). He described applying gentle traction to the retracted nerve stumps, suturing using an alcohol disinfected needle, and finally, insulating the sutured segment with a mixture of oils. The injured limb was later immobilized to prevent damage to the suture (Artico et al., 1996; Ngeow, 2010). The whole procedure closely resembles modern surgical protocol, which includes disinfection, appropriate identification of injured nerve trunk, suturing, and wound immobilization (Artico et al., 1996; Ngeow, 2010). Over the years, different procedures have been developed to boost intrinsic neuronal growth properties. In particular, physical therapies have evolved, such as treadmill training, resistance training, and swimming, which aim to maintain muscle strength, relieve pain, and enhance functional recovery (Bonetti et al., 2011). Electrical stimulations have been also developed to facilitate rehabilitation after nerve injuries in animal models and human patients (Nix and Hopf, 1983; Ahlborn et al., 2007; Gordon et al., 2009b). In addition, pharmacological elevation of cAMP and overexpression of neuronal growth-associated genes such as GAP-43 or CAP-23 have been reported to accelerate axon outgrowth and promote robust regeneration (Caroni et al., 1997; Bomze et al., 2001; Cai et al., 2001).

Despite the technological advances and extensive research on nerve regeneration, the functional outcome after nerve injury and repair are generally still insufficient, particularly when sensory functions are considered (Dahlin et al., 2009). The failure of functional recovery after peripheral nerve injuries can be explained by various factors. It can result from the damage to the neuronal cell body due to axotomy, retrograde degeneration (Krarup et al., 2002; Schmidhammer et al., 2007), and neuronal loss (Witzel et al., 2005; Navarro et al., 2007). Since peripheral nerve injuries inherently involve inflammatory component, regenerative attempts over long distance are always impeded by connective tissue scarring (Deumens et al., 2010; Ngeow, 2010). Underlying diseases, such as diabetic generalized neuropathy, may impede axonal regeneration as well (Stoll and Muller, 1999). Potentially, failure of functional recovery may also result from the poor specificity of re-innervation. The selectivity of axon-target reconnection plays an important role in the recovery of function after nerve injury and regeneration. During the process of nerve regeneration, several sprouts emerge from each parent axon (Witzel et al., 2005). When axons reconnect with the appropriate peripheral tissue, misdirected axonal sprouts are withdrawn gradually. The pruning of supernumerary axonal sprouts helps to refine the selectivity of axon-target reconnection. However, the later refinement of distal reconnection and the re-innervation of targets are often far from adequate. When inappropriate distal reconnection is established, disturbed sensory localization or limited fine motor control is expected to follow (English et al., 2011). The failure of functional recovery may also stem from the slow rate of axonal growth. At an average rate of 1–3 mm/day for axonal regeneration in mammals, weeks or even months could be anticipated for signs of functional recovery (Gordon et al., 2009a). The long time period required for the regeneration is responsible for concomitant denervation atrophy of the target tissue (Gordon et al., 2009a). If the denervated skeletal muscles are replaced by adipose tissue, despite the fact that peripheral axons can regenerate through the injury site, functional recovery is severely compromised.

Currently, no medical treatment can overcome the limitations in axonal regeneration, and ensure the recovery of normal sensory and motor functions following nerve trauma, and it is a general consensus that the standard treatment options have reached a plateau (Navarro et al., 2007). Therefore, new therapeutic intervention strategies for peripheral nerve repair are critically needed. A better understanding of the molecular and cellular mechanisms involved in successful axon regeneration and appropriate target re-innervation would be most helpful in developing new therapeutic applications.

## Endogenous mechanisms that support peripheral axon regeneration

Nerve injuries are powerful stimuli that lead to profound cellular responses. Following an injury, axons and myelin sheaths distal to the lesion site are degraded by a process of Wallerian degeneration (Glass, 2004; Makwana and Raivich, 2005). Myelin breaks down to vesicles, resulting in the collapse of the myelin sheath (Ngeow, 2010). Schwann cell cytoplasm withdraws from the myelin vesicles and significantly decreases the synthesis of myelin lipids and proteins between 12 and 48 h post the injury (Ngeow, 2010). Such damage increases the permeability of the blood-nerve barrier, which allows for the recruitment of macrophages to the site of the injured nerve. Infiltrating macrophages and injury-activated Schwann cells phagocytize the degenerative end products (Stoll and Muller, 1999). Wallerian degeneration takes place during the first few days. During this stage, elimination of myelin sheaths is important, because it clears the regeneration-inhibitory factors associated with myelin (Skaper, 2005; Raivich and Makwana, 2007). At the same time, retrograde degeneration also takes place at a short segment of the proximal nerve stump. The remaining axons in the proximal nerve also exhibit a reduction in diameter, followed by chromatolysis at the neuron soma and dendritic arbor retraction (Hanz and Fainzilber, 2006; Navarro et al., 2007). Chromatolysis, characterized by the loss and dispersion of the Nissl bodies, reflects a reactive alteration in neuronal biochemistry and function, when the neuronal cells shift their functions from the synthesis of proteins required for neurotransmission to those required for regenerative axon growth (Deumens et al., 2010).

Loss of axonal contact also triggers dedifferentiation and proliferation of Schwann cells in the distal nerve (Karanth et al., 2006; Navarro et al., 2007). Proliferated Schwann cells line up in bands of Bungner, which later provide support for regenerating axons (Geuna et al., 2009). Schwann cells not only pave a path for regenerating axons to grow, they also attract injured neurons by secreting neurotrophic factors, such as nerve growth factor (NGF) (Ngeow, 2010). Proximal to the lesion, fine sprouts emerge (Witzel et al., 2005) and using distal endoneurial tube as a guiding structure, elongate in association with Schwann cells toward targets (Stoll and Muller, 1999; Navarro et al., 2007). In the absence of a guiding structure, regenerating axons may form neuroma, a growth composed of immature axonal sprouts and connective tissue.

Finally, regenerated axons reconnect with target peripheral tissue. Because several sprouts emerge from each parent axon, supernumerary axonal sprouts will be withdrawn gradually during the maturation of the nerve fiber (Witzel et al., 2005; Navarro et al., 2007). The regenerated axons will have smaller caliber and with shorter internodes than normal nerve structures (Geuna et al., 2009). For successful regeneration, the axons are expected to replace the distal nerve segment lost during degeneration. However, more often than not, the regenerated axons do not innervate target tissues adequately or relay information from sensory receptor accurately, reducing the recovery of motor and sensory functions, especially when the lesion is severe (Choi et al., 2005; Bannerman and James, 2009). Usually, after nerve injury and repair, the diameter of regenerated axons, as well as their conduction velocity and excitability remain below normal levels for a long time. Consequently, this results in incomplete and inadequate functional recovery of reinnervated organs (Van Meeteren et al., 1997; Xiao et al., 2007).

## Molecular bases of peripheral nerve regeneration

**Figure 1:**
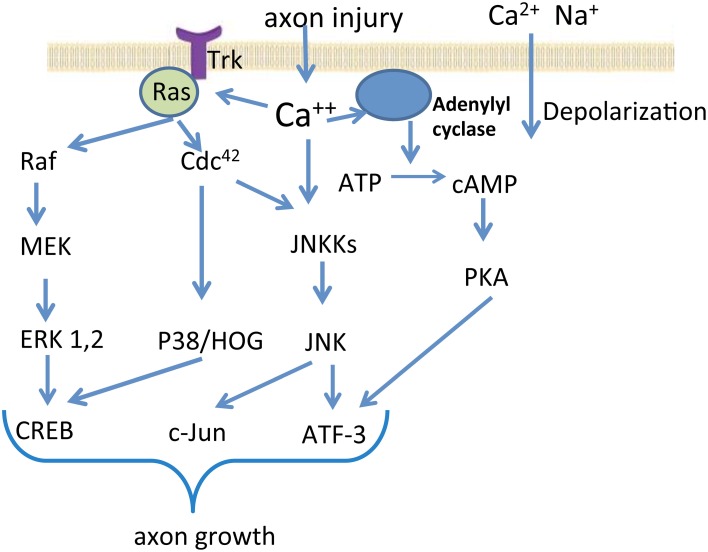
**Signal transduction in neuronal cells after nerve injury.** The schematic diagram depicts several important signaling pathways activated by axon injury. Cellular injury induces sodium and calcium influx, which lead to depolarization. Among many other processes, the elevated intracellular calcium concentration leads to the activation of protein kinase pathways such as the MAPK Erk1 and Erk2, JNK, and P38 kinase. Downstream events influenced by axotomy-activated kinases include up-regulation or activation of several transcription factors. The modifications in the activity of transcription factors result in characteristic changes of gene expression in the injured and regenerating neurons.

Previous studies have demonstrated that to initiate a regenerative response to injury in the peripheral nerve system (PNS), the neuron must shift its physiology from synaptic transmission and maintenance of structure to the growth of the axon (Benowitz and Yin, 2007). A sequence of molecular responses would take place in response to injury for the successful nerve regeneration and recovery of function (Figure 1). After nerve lesion, calcium and sodium ions influx into axoplasm through the ruptured plasmatic membrane, generating high frequency burst of action potentials at the lesioned site (Makwana and Raivich, 2005; Navarro et al., 2007). This first signal promotes an influx of calcium through voltage-dependent ion channels, and leads to the activation of several protein kinase pathways, including: calcium/calmodulin dependent kinase 2 (CMAK2), protein kinase A (PKA), protein kinase C (PKC), and mitogen-activated protein kinase (MAPK), such as Erk1 and Erk2, c-jun N-terminal kinase (JNK) and P38 kinase (Makwana and Raivich, 2005; Raivich and Makwana, 2007). During the second phase of signaling, these activated proteins, termed “positive injury signals,” incorporate the retrograde transport system for trafficking back to the cell body from the injured site and induce several signaling pathways genes (Hanz and Fainzilber, 2006). Several transcription factors have been identified as the mediators in the regulation of gene expression. The change in activity of transcription factors is considered the downstream event influenced by axotomy-activated protein kinases (Dahlin et al., 2009). The activation of transcription factor cAMP responsive element binding protein (CREB) has been demonstrated in early stages after injury (Miletic et al., 2004b). Phosphorylation of CREB can be mediated by multiple protein kinase pathways through activation of tyrosine kinase receptors (Trk) (Miletic et al., 2004a). Two other transcription factors induced by nerve injury are ATF-3 and c-Jun. c-Jun up-regulation and phosphorylation is induced by activated JNK, leading to the formation of activating protein 1 (AP-1) complexes. JNK pathways and the Erk1/2 pathways also show cross-talk coordinated by MEKK1 in PC12 cells (Waetzig and Herdegen, 2005). ATF-3 is induced in all dorsal root ganglia (DRG) neurons after peripheral axotomy, which makes it a reliable nerve injury marker. Inhibition of JNK reduces ATF-3 expression, which impairs nerve regeneration (Dahlin et al., 2009). The modifications in the activity of transcription factors result in characteristic changes of gene expression. Hundreds of genes have been found differentially expressed after a nerve injury, including genes encoding for transcription factors, cytoskeletal proteins, cell adhesion and guidance molecules, trophic factors and receptors, cytokines, neuropeptides and neurotransmitter synthesizing enzymes, ion channels, and membrane transporters (Navarro et al., 2007; Raivich and Makwana, 2007; Dziennis and Alkayed, 2008; Hou et al., 2008). The changes in gene expression support the formation of new growth cones and elongation of the regenerating axon, leading to nerve regeneration. The changes in the gene expression that promote nerve regeneration could also be induces by factors released by non-neuronal cells (Zigmond, 2012). One example is interleukin (IL)-6, a member of cytokines family referred to as the glycoprotein (gp) 130 family. Injury induced IL-6 activates the phosphorylation of STAT3, and the expression of a set of responsive genes (Heinrich et al., 2003). While the release of gp130 cytokines promotes nerve regeneration, IL-6 knockout impairs the normal functional recovery after sciatic nerve injury (Zhong et al., 1999; Yang et al., 2012).

## Post-transcriptional regulation of gene expression

The discoveries of micro RNAs (miRNAs) and RNA interference (RNAi) have revolutionized our understanding of post-transcriptional regulation of gene expression and have provided powerful tools for targeted gene silencing.

The phenomenon of post-transcriptional regulation of gene expression by small RNAs was first observed in petunia. When an exogenous RNA sequence was introduced into petunia, instead of being translated into protein, it “silenced” the endogenous homologous gene's expression (Napoli et al., 1990). This gene-silencing phenomenon was then characterized in *Caenorhabditis elegans* by Andrew Fire and Craig Mello, which they termed “RNAi” (Fire et al., 1998). Subsequent studies have also shown that long double-stranded RNAs (dsRNAs) can induce a sequence-specific inhibition of gene expression in a number of invertebrates, whereas shorter dsRNA, termed small interfering RNAs (siRNAs), are required to induce highly specific gene silencing in mammalian cells (Elbashir et al., 2001).

RNAi is an evolutionary conserved mechanism to selectively suppress gene expression (Filipowicz et al., 2008). It was originally recognized as a defensive response to foreign nucleic acids. Eukaryotic cells infected by viruses can process the dsRNA carried by viruses into siRNAs, which bind to and cause degradation of matched messenger RNA (mRNA), preventing the synthesis of protein necessary for viral replication. RNAi also protect the eukaryotic genome from endogenous transposable elements, and it was later demonstrated that RNAi is required for normal development (Saugstad, 2010). Both exogenous double-stranded siRNA and endogenous single-stranded miRNA can initiate and utilize the same RNAi machinery to produce gene silencing (Rana, 2007).

## miRNA biogenesis and RISC assembly

**Figure 2:**
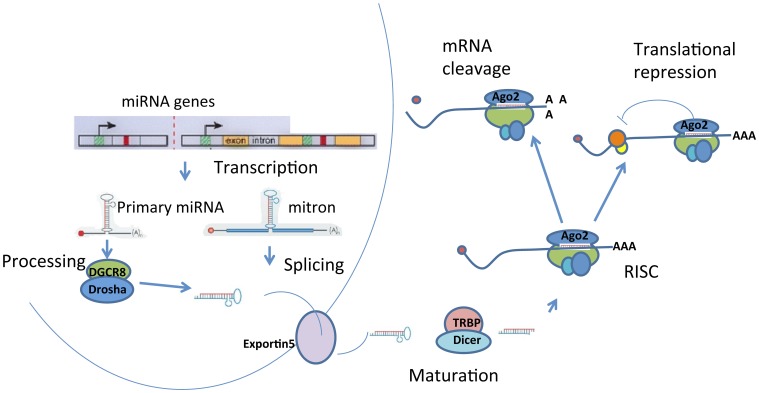
**Biogenesis of miRNAs.** miRNAs are processed from precursor molecules, which are either transcripts from independent miRNA genes (pri-miRNA) or are a portion of introns of protein coding transcripts (mitron). The precursor molecules are excised into pre-miRNA with a hairpin structure. The final processing of pre-miRNA by *Dicer* yields miRNA duplex. One strand of the duplex is degraded and the remaining mature miRNA binds to Argonaute proteins to form RNA-induced silencing complexes (RISCs). miRNAs target sequences within messenger RNAs, causing repression of translation and subsequent degradation or storage of mRNAs in P-bodies.

miRNAs are endogenous non-coding 21–23 nucleotide small RNA molecules that regulate gene expression by binding to the 3′-untranslated region of target mRNAs, leading to their translational inhibition or degradation (Filipowicz et al., 2008; Carthew and Sontheimer, 2009). miRNAs are encoded in genomic DNA, located either in the introns of protein-coding genes or as independent entities (Figure 2). miRNA genes are first transcribed by RNA polymerase II into primary miRNA (pri-miRNAs). A single pri-miRNAs often contains sequences for several different miRNAs folded into imperfectly base-paired hairpin structures. Primary miRNAs are cleaved by enzymes, such as Drosha and DGCR8, into ~70 nucleotide hairpins known as precursor miRNAs (pre-miRNAs). Alternatively, miRNA transcription may occur from the introns of protein-coding genes, called “mirtron” or “mitron.” Mitrons are spliced out from premature mRNA to form pre-miRNAs (Sibley et al., 2012), which bypass the Drosha/DGCR8 enzyme complex. Regardless of the initial source, pre-miRNAs are then transported into the cytoplasm by Exportin-5 where they are further processed by the endoribonuclease called *Dicer*. In mammals, *Dicer* forms a complex with human immunodeficiency virus (HIV) transactivating response RNA (TAR) binding protein (TRBP) and in *D. melanogaster Dicer* complexes with Loquacious (Bernstein et al., 2001; Lee et al., 2003; Filipowicz et al., 2008). TRBP interacts with PACT (a protein activator of the interferon-induced protein kinase, PKR) to mediate RNAi and micro-RNA processing (Kok et al., 2007). The products of *Dicer* processing form miRNA duplexes with protruding 2-nucleotide 3′ end. The strand with the 5′ terminus located at the thermodynamically less-stable end of the duplex is usually selected to function as a guide strand for the mature miRNA, while the opposite strand (or “passenger”) is degraded. Occasionally, both strands give rise to mature miRNA (Filipowicz et al., 2008). They are designated as miR-X and miR-X^*^, with the less predominately expressed transcript indicated by an asterisk (Saugstad, 2010).

The mature miRNA target specific mRNAs to either cause degradation of the mRNA or inhibit protein translation via RNA induced silencing complex (RISC), a ribonucleoprotein complex associated with miRNA. Although, assembly of RISC is a very dynamic process and is not well understood, proteins of Argonaute (Ago) family are considered the most important components of RISC (Peters and Meister, 2007; Filipowicz et al., 2008). The number of Ago proteins differs between species. For example, humans have eight Ago proteins, *Drosophila* have five, and *C. elegans* express 27 Ago proteins (Sasaki et al., 2003; Peters and Meister, 2007). In mammals, only Ago2 can cleave mRNA at the center of the siRNA-mRNA duplex. The significance of Ago2 in the RNAi pathway is also evidenced by the significant reduction in RNAi function after Ago2 knockdown (Hammond et al., 2001).

Apart from Ago proteins, RISC contains other regulatory factors and effectors that mediate inhibitory function. The fragile X mental retardation protein (FMRP) is one of the conclusively identified subunits. Both miRNA and siRNA can co-immunoprecipitate with FMRP. The FMRP belongs to the fragile X gene family, which encodes three different proteins: FMRP, FXR1, and FXR2. The loss-of-function mutations in the FMRP gene results in fragile X syndrome (FXS), whereas the function of FXR1 and FXR2 remains unknown. However, it is suspected that all three fragile X protein functions similarly in regulating mRNA stability by binding with ribosomes, and FXR1 and FXR2 has the potential to associate with RISC as well (Siomi et al., 2004). Other identified components of RISC include Gemin3 and Gemin4, which are also part of the survival of motor neurons (SMN) complex. The SMN complex plays a critical role in the assembly of diverse ribonucleoprotein complexes in the nervous system. The functions of Gemin3 and Gemin4 are speculated to be involved in target mRNA recognition and translational repression (Dostie et al., 2003; Battle et al., 2007). RISC also recruits P-100. Some studies have demonstrated that P-100 may be a nuclease in RISC since it showed ribonuclease activity (Sundstrom et al., 2009).

## The miRNA target mRNA interaction

miRNAs in RISC form complementary base pairs with mRNAs within their 3′-UTR, leading to their translational inhibition or degradation (Carthew and Sontheimer, 2009). In plants miRNAs usually have a complete complementarity to target mRNA, which triggers mRNA degradation. In animals, miRNAs do not have a complete match to mRNA and therefore miRNA-mRNA binding causes translational block. The translational repression is triggered by binding of the miRNA seed region to mRNA. The primary rule for mRNA targeting is the perfect base pairing of the seed region, which is located at the miRNA nucleotides 2–8. Perfect and contiguous Watson–Crick base pairing at this limited region links miRNA with target mRNA (Filipowicz et al., 2008). The second rule requires a mismatch to be present in the central region of the miRNA-mRNA duplex. The bulge generated by the mismatch precludes the Ago-2 mediated endonucleolytic cleavage of mRNA (Filipowicz et al., 2008). Some base pairing at the other half side of miRNAs, especially the 13–16 nucleotides, would stabilize the binding between miRNA and mRNA (Grimson et al., 2007). Multiple miRNA binding sites within the mRNA 3′ UTR can improve the silencing efficiency (Doench and Sharp, 2004). A position of binding site close to the poly(A) tail or the termination codon increases the accessibility of mRNA to RISC (Grimson et al., 2007). Interactions between proteins bound to miRNA or mRNAs can also influence target selection and efficiency of repression. However, because of the incomplete match between miRNAs to mRNAs in animals, each miRNA may potentially target numerous genes, which makes the prediction of miRNAs targets complicated and usually insufficient (Ritchie et al., 2009).

## P-bodies as a sites for miRNA mediated translational repression and degradation

Several independent studies have shown that Ago proteins may interact with the RNA-binding GW182 proteins (Sen and Blau, 2005; Eulalio et al., 2009; Takimoto et al., 2009). Subsequent observations revealed that GW182-containing foci, known as GW bodies (GWBs), coincide with mRNA-processing bodies or P-bodies (Sen and Blau, 2005; Eulalio et al., 2009; Takimoto et al., 2009). The cytoplasmic foci, termed P-bodies, serve as the sites where mRNAs undergo degradation and storage (Ding and Han, 2007). In eukaryotes, mRNA degradation can follow two pathways that are initiated by a gradual shortening of the mRNA poly(A) tail with deadenylases. In the first pathway, following deadenylation, mRNAs are exonucleolytically digested from 3′ to 5′, which is catalyzed by the exosome (Eulalio et al., 2007b; Filipowicz et al., 2008). In the second pathway, the cap structures at 5′ end are removed by decapping enzymes after deadenylation, followed by 5′ to 3′ degradation catalyzed by exonuclease. The decapping enzymes and decapping coactivator, including DCP1 and DCP2, as well as exonuclease XRN1, colocalize in P-bodies. Together they form the decay machinery, which destabilizes mRNAs in P-bodies (Eulalio et al., 2007a). The knockdown of the decay machinery components prevents miRNA-mediated degradation (Filipowicz et al., 2008). Additional P-bodies components include: decapping activators RCK/p54 and Pat1, translational repressor eIF4E-transporter (4E-T), and RAP55. RAP55 responds to stress and has a putative role in translation regulation (Eulalio et al., 2007b).

In addition to mRNA degradation, an alternative mechanism to repress target mRNA expression is through translation inhibition. Translation requires the participation of several initiation factors, many of which are multiprotein complexes. Initiation of translation starts with the binding between mRNA 7-methylguanosine cap and eukaryotic translation initiation factor (eIF) 4E. When miRNA is bound to the mRNA 3′ UTR, Ago proteins interact with the 7-methylguanosine cap of the mRNA. The association of Ago, instead of eIF4E, with 7-methylguanosine cap prevents effective recruitment of ribosomes and therefore blocks the initiation of translation (Kiriakidou et al., 2007). Furthermore, studies have also shown that multiple miRNA binding sites at the 3′ UTR increase the likelihood of Ago association with 7-methylguanosine cap, thereby enhancing the translational repression (Filipowicz et al., 2008). Association of Ago with 7-methylguanosine cap also disrupts the 3D structure of the mRNA, and possibly makes the poly (A) tail more vulnerable to exonucleolytic activity (Eulalio et al., 2007b).

Targeting translation initiation is not the only mechanism miRNAs use to inhibit mRNA translation. The inhibition of translation occurs at the post-initiation phase as well. Studies have shown that mRNAs targeted by miRNAs remain associated with polysomes, despite a strong reduction in the protein expression level (Filipowicz et al., 2008). miRNAs are speculated to slow elongation and induce ribosome drop off from mRNA. How miRNA could modulate the elongation or terminate the translation of mRNA remains unclear.

Recent findings indicate that under certain conditions mRNAs sequestered into P-bodies can be freed when cells respond to a subsequent stress. The mRNAs released from P-bodies are then recruited to the polysome and translation can be resumed (Saugstad, 2010). The ability of miRNA to disengage from repressed mRNA makes miRNA regulation more dynamic and wide-ranging. One example in neuronal cells is miR-134 mediated repression of LIMK1, a protein kinase that is important for the development of dendritic spines. In response to extracellular stimuli, miR-134 mediated repression of LIMK1 is relieved at dendritic spines (Schratt et al., 2006). This observation further suggests that miRNA regulated mRNA translation is probably an important regulator of gene expression in response to synaptic activity.

## Functional significance of miRNAs

Bioinformatic predictions indicate that mammalian miRNAs can regulate at least 30% of all protein coding genes (Filipowicz et al., 2008). Therefore, it is no surprise that miRNA based regulations are involved in many cellular processes. miRNA plays diverse roles in cell differentiation, proliferation (Stefani and Slack, 2008) metabolism (Wang et al., 2011), and signal transduction (McCoy, 2012).

## miRNAs in neural development

Early studies revealed that decreasing miRNA production by *Dicer* gene ablation induced embryonic lethality, suggesting miRNA plays a critical role in normal fetal development (Bernstein et al., 2003). The conditional *Dicer* knockout approaches showed pivotal role of miRNA in neural development. *Dicer* deletion in neocortex disrupted differentiation of newborn neurons and results in neuronal cell death (De Pietri Tonelli et al., 2008). *Dicer* ablation in hippocampus at different embryonic time points resulted in abnormal hippocampal morphology, and affected the number of hippocampal progenitors due to altered proliferation and increased apoptosis (Li et al., 2011). *Dicer*-null neural stem cells were incapable of generating either glial or neuronal progeny, which blocked the differentiation (Andersson et al., 2010). Conditional knockouts for Dicer also resulted in the malformation of the midbrain and cerebellum, and failure of neural crest and dopaminergic differentiation in mice (Huang et al., 2010).

## miRNAs as effectors in neurological disorders

A number of studies have shown a correlation between neurological diseases and the alteration of miRNA biogenesis. Although no conclusion can be made that the altered expression of miRNAs is a consequence or the cause of neurological disorders, some studies showed the change in miRNA expression prior to the onset of the disease (Wang et al., 2008b). This raises the possibility that restoring the expressing level of specific miRNAs could prevent the pathological development of the diseases.

Mouse models and human samples have both implicated altered miRNAs in the Alzheimer's disease (AD), particularly with respect to the regulation of β-amyloid precursor protein converting enzyme1 (BACE1) (Hutchison et al., 2009). BACE1 was shown to be targeted by miR-458-5p, and an endogenous natural BACE-antisense competes with miR-485-5p for the binding on BACE1 mRNAs. In AD patients, BACE-antisense appeared to be up-regulated while miR-485-5p was down-regulated in cortex and hippocampus. Therefore, the down-regulation of BACE1 translation was blocked, which stimulated the formation of amyloid-β-peptide plaques (Faghihi et al., 2010). miR-29 was found to regulate BACE1 expression *in vitro*, and decreased expression of miR-29a and miR-29b was observed in AD brains (Hebert and De Strooper, 2007; Hebert et al., 2008). In addition, miR-107 significantly decreases at early stage of AD, and has multiple predicted binding sites on BACE1 (Wang et al., 2008b). In mouse models, miR-298 and miR-328 can also target BACE1 mRNA, and *in vitro* studies confirm the regulation of BACE1 protein expression by these miRNAs (Boissonneault et al., 2009). Increased expression of miRNAs, such as miR-9, miR-125b, miR-138, and miR-146a have also been observed in AD brains (Saugstad, 2010; Olde Loohuis et al., 2012).

The pathological development of Parkinson's disease (PD) is under the control of miRNAs as well. Conditional knockout of *Dicer* in dopaminergic neurons results in the loss of dopaminergic neurons and impaired locomotion, mimicking the phenotype of PD patients (Olde Loohuis et al., 2012). Gene screen of PD patients identified a disruption in the binding site of miR-433 in the 3′ UTR of the fibroblast growth factor 20 (FGF20) gene. This disruption leads to an increased expression of FGF20 and a subsequent increase of α-synuclein expression, which is correlated with cytotoxicity associated with PD (Wang et al., 2008a). Increased expression of α-synuclein may also result from insufficient expression of miR-7 in neurotoxin model of PD (Junn et al., 2009). Another report found a significant decrease of miR-133b expression in PD patients. Additional studies revealed that miR-133b is specifically expressed in midbrain dopaminergic neurons, and targets the transcription factor pituitary homeobox 3 (Pitx3). Lack of miR-133 disrupted midbrain dopaminergic maturation and function (Kim et al., 2007).

Studies on miRNA expression in Huntington's disease (HD) brains revealed dysregulated expression of several miRNAs in both mouse models and human patients (Hutchison et al., 2009). HD is related to abnormal activation of the transcription factor REST. When Huntington protein cannot bind REST, REST can freely translocate to the nucleus and repress neuronal gene expression. Studies showed that several miRNAs with REST binding sites or REST cofactor binding sites are decreased in human HD, including miR-9 and miR-9^*^, which target REST and co-REST, respectively (Packer et al., 2008). miR-124, which plays a role in maintaining neuronal identity through targeting PtBP-1is also decreased in HD patients (Cao et al., 2007).

Interestingly, intellectual disability syndromes and mental diseases appear to be influenced by miRNA expression as well. Studies in schizophrenia patients revealed a significant increase in global miRNA expression (Beveridge et al., 2010). miR-160b, 30b, and 181b were significantly up-regulated in the frontal cortex of schizophrenia patients (Kim et al., 2010; Santarelli et al., 2011). NMDA-regulated miRNA miR-132 was significantly down-regulated in the prefrontal cortical tissue from schizophrenia patients (Miller et al., 2012). Twenty-eight miRNAs are differently expressed in the brains of autistic patients, and the predicted targets of dysregulated miRNAs include Neurexin and SHANK3, which are known genetic causes of autism (Abu-Elneel et al., 2008). In another study, differential expressions of nine miRNAs were observed in autism samples in growing lymphoblastoid cell lines (Talebizadeh et al., 2008). These studies reveal a subset of brain-related microRNAs implicated in schizophrenia and autism.

## miRNAs located in neurons

In neurons, the functions of individual miRNAs are just beginning to emerge. To date, seven miRNAs have been identified to be specifically expressed in mammalian brain, which includes miR-9, miR-124a, miR-124b, miR-135, miR-153, miR-183, and miR-219; suggesting their unique regulatory roles in brain function (Sempere et al., 2004). Functional analysis showed that transfection of brain-specific miR-124 into HeLa cells is sufficient to shift the gene expression profile toward neuronal-like phenotype (Lim et al., 2005). One direct target of miR-124 is small c-terminal domain phosphatase 1, an anti-neural factor of REST/SCP1 pathway. Suppression of small c-terminal domain phosphatase 1 induces neurogenesis during brain development (Visvanathan et al., 2007). MiR-124 also represses the expression of SRY-box transcription factor Sox9. This repression promotes adult neurogenesis in the subventricular zone stem cell niche (Cheng et al., 2009). MiR-124 also regulates early neurogenesis in the forebrain and optic vesicle by targeting NeuroD1 (Liu et al., 2011). More recently, miRNA expression profiles have been identified within brain regions, such as the cortex and hippocampus. It was found that the expression of let-7g, miR-92b, miR-146b, miR-330^*^, and miR-394 were significantly higher in rat hippocampus than in cortex (Olde Loohuis et al., 2012). The specificity in miRNA expression further suggested the cell or tissue specific functions of miRNAs. For example, miR-449 has been identified in the choroid plexus, the area in the brain ventricle that is responsible for the production of cerebrospinal fluid. Transcription factor E2f5, which regulates cerebrospinal fluid production is targeted by miR-449 (Redshaw et al., 2009).

## miRNAs in axons and dendrites

Subcellular localization of miRNAs within neuronal dendrites and axons has been shown in recent studies. With laser capture microdissection, RNA populations from dendrites and cell bodies were acquired. RT-qPCR analysis revealed that most miRNAs distributed with a gradient decrease from soma into the dendrites. A few miRNAs, such as miR-26a, miR-26b, and miR-292-5p, are enriched in dendrites (Kye et al., 2007). Enrichment of precursor miRNAs has been observed in dendrites as well. These dendritically enriched precursor miRNAs show a distinct structure which may allow for binding to proteins that mediate dendritic transport (Smalheiser, 2008). The significant correlation between precursor and mature miRNAs enrichments suggests that precursor miRNAs may be processed locally in dendrites. The identification of *Dicer* at postsynaptic densities further supports this hypothesis (Lugli et al., 2005, 2008). It has been widely accepted that local protein synthesis in distal domains of neuron has a key role in synaptic formation, synaptic plasticity, and axonal regeneration (Schacher and Wu, 2002; Hanz et al., 2003). Regulation of local mRNA translation can alter the synaptic protein expression upon stimulations. One theory for miRNA-based regulation of local protein synthesis suggests that precursor miRNAs are predominantly associated with postsynaptic densities. Upon stimulation neuronal-mediated calcium influxes activate proteases, such as calpain, resulting in the release of *Dicer* from the postsynaptic density. The newly freed *Dicer* processes precursor miRNAs into mature miRNAs, which then incorporate into RISC and inhibit target mRNA translation. Meanwhile, activated proteases can also degrade components of RISC. mRNAs that are important for synaptic plasticity can be released from RISC and selectively enter the polysome compartment where they will resume the initiation of translation. This local translational regulatory model provides a mechanism that meets the requirements for both increased and decreased protein synthesis (Saugstad, 2010).

A small number of miRNAs involved in synaptic morphogenesis and plasticity have been identified through recent functional analysis. miR-138 is localized in dendrites of rat hippocampal neurons, and it inhibits dendrite spine growth through activating the Rho signaling pathway. miR-138 targets acyl protein thioesterase 1 (APT1), which in turn catalyzes the membrane association of Gα12/13. The membrane association of small G protein subunit Gα13 has been shown to be involved in the activation of RhoA signaling pathway (Siegel et al., 2009). In contrary, miR-132 expression enhances dendrite morphogenesis in hippocampal neurons. miR-132 represses the expression of the Rho GTPase-activating protein (GAP) p250. p250GAP regulates spine formation by modulating Rac1 and RhoA activities. Therefore, miR-132 expression is related to P250GAP. Introduction of miR-132 showed the same effect on dendritic spine phenotype as p250GAP knockout, which results in increased spine density and size (Olde Loohuis et al., 2012). miR-134 is an example of neurotrophic control of dendritic spine plasticity through a miRNA mechanism. Localization of miR-134 to dendrites negatively regulates the size of dendritic spine by inhibiting the expression of LIM domain kinase 1(Limk1), neurogenic differentiation factor 2 (NeuroD2), and disks large homolog2 (DLG2). Exposure to brain-derived neurotrophic factor (BDNF) relieves the inhibition in spine development by miR-134, and increases the expression of Limk1 protein. Limk1 regulates actin filament dynamics, thereby controlling cytoskeletal reorganization, and promoting new spine outgrowth (Schratt et al., 2006). The involvement of miRNAs in synaptic plasticity is further confirmed with the changes of miRNA expressions after long-term potentiation.

Compared with the studies on dendritic miRNAs, there is less understanding in the role of miRNA in axonal functions. However, the studies investigating axonal miRNA pathway are growing. Pure axonal miRNAs have been obtained from superior cervical ganglia neurons cultured in compartmentalized Campenot cell culture chambers. In total, 130 mature miRNAs have been detected in distal axons, and a few, such as, miR-15b, miR-16, and miR-221, were highly enriched in axons (Natera-Naranjo et al., 2010). miRNA machinery components, including Dicer, Ago proteins, and a fragile-X mental retardation protein (FMRP), have been found to be localized in developing axons and growth cones in RNA granule-like structures (Hengst et al., 2006). Although direct evidence for miRNA-mediated suppression of axonal mRNA translation has not been demonstrated, the involvement of the miRNA pathway in the regulation of axonal function is suggested by the functional activity of miRNA machinery in axons (Hengst et al., 2006). The existence of protein components of RISC was shown in sciatic nerves. Transfection of siRNA against neuronal β-tubulin into axons initiated the formation of RISC and the suppressions of target gene (Murashov et al., 2007b).

## Injury induced miRNA expression after peripheral nerve injury

**Figure 3:**
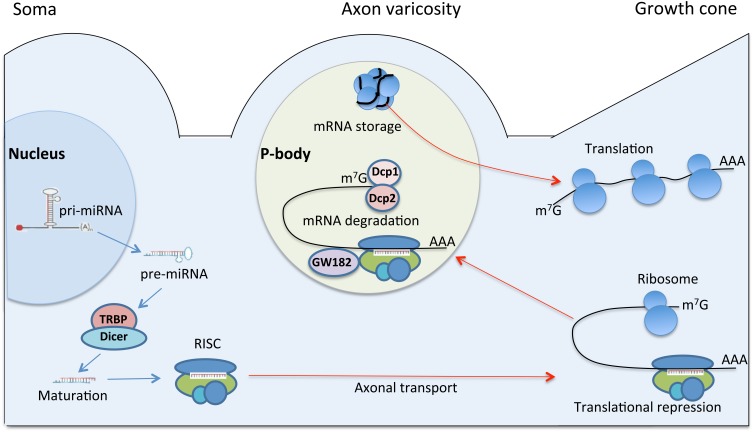
**A model for regulation of axonal protein synthesis by miRNA in regeneration.** The illustration depicts neuronal cell body, axon varicosity and the growth cone. Pri-miRNA is transcribed in the nucleus and transported to cytoplasm as pre-miRNA. In the cytoplasm, pre-miRNA is processed by Dicer/TRBP complex into mature miRNA, which is loaded onto RISC. RISC is a nucleoprotein complex, which may be transported to distal regions of neuron including the growth cone. In the growth cone RISC may regulate protein translation by silencing specific mRNAs and causing translational repression. The repressed mRNAs may translocate into P-body, a specific foci, primarily localized to axon varicosities. Axon varicosity is a place of organelle accumulation (Bennett and Muschol, 2009), clustering of excess growth resources (Malkinson and Spira, 2010), mRNA concentration (Lee and Hollenbeck, 2003), and P-body accumulation (Wu et al., 2011). The P-body machinery localized to varicosities may regulate the growth resources by managing mRNAs pool. In P-body, mRNAs targeted for destruction are associated with decapping enzymes Dcp1 and Dcp2, as well as GW182. Translationally repressed mRNA might be also stored in P-body. Upon changes in cellular conditions and stimuli, some of the stored mRNAs can re-enter the translation pathway (Rana, 2007).

The involvement of the miRNA biosynthetic machinery in the regulation of intra-axonal local protein synthesis after injury was confirmed by the injury-regulated expression of biosynthetic enzymes, including components of RISC and P-bodies (Wu et al., 2011). In particular, researchers observed an increase in the number and the size of P-bodies in the regenerating DRG axons after conditioning sciatic nerve lesion. The P-bodies were primarily localized to the axon varicosities. The physiological role of axon varicosities has been associated with places of organelle accumulation and sequestration (Bennett and Muschol, 2009), and clustering of excess growth resources (Malkinson and Spira, 2010). The varicosities were also identified as the sites of mRNA concentration and protein synthesis (Lee and Hollenbeck, 2003). Interestingly, the number of varicosities in the regenerating DRG neurons was markedly higher than in naïve. Taken together, these data suggest that P-body machinery localized to varicosities could regulate the growth resources by managing mRNA pool (Wu et al., 2011) (Figure 3).

Further studies showed that inducible deletion of *Dicer* impaired nerve regeneration according to functional behavioral tests, electrophysiological, and histological analyses in a mouse model of peripheral nerve crush. In addition, *Dicer*-deficient neurons failed to regenerate axons in DRG cultures after conditioning sciatic nerve lesion. Thus, this study demonstrated that the intact *Dicer*-dependent miRNA pathway is critical for the successful functional recovery *in vivo* and the regenerative axonogenesis *in vitro* (Wu et al., 2012).

Furthermore, a group of miRNAs that are specifically expressed in an injury-regulated pattern in the regenerating sciatic nerves and DRG were identified by miRNA microarrays and confirmed by qPCR (Strickland et al., 2011; Wu et al., 2011). The most upregulated miRNA, miR-21 has the ability to promote axon growth in adult DRG neurons through targeting SPRY2 (Strickland et al., 2011). Overexpression of miR-21 also protects against ischemic neuronal death, probably mediated by its downregulation of FASLG, an important cell death-inducing ligand (Buller et al., 2010). The upregulated miR-29b in sciatic nerves during nerve regeneration exhibited neuronal protective effects (Kole et al., 2011). It was shown that miR-29b functions as a novel inhibitor of neuronal apoptosis by targeting multiple proapoptotic BH3-3 gene family (Kole et al., 2011). Some of the upregulated miRNAs have injury-induced expression patterns in CNS as well, such as miR-211 and miR-142-5P. Their expression levels were increased after brain injury or spinal injury, respectively (Lei et al., 2009; Liu et al., 2009). The downregulated miRNA, miR-145, has been shown to inhibit neurite outgrowth *in vitro* with robo2 and srGAP2 validated as its potential target genes (Zhang et al., 2011). Surprisingly, some miRNAs showed a decrease in their expression level in the sciatic nerve microarray analysis correlating with a positive effect on axon outgrowth, such as miR-124a and miR-133. miR-124a decreased its level in sciatic nerves after crush, however, it was required for hippocampal axonogenesis through Lhx2 suppression, which also prevents apoptosis in the developing retina (Sanuki et al., 2011). In zebra fish, miR-133 has been shown to promote tissue regeneration by targeting the small GTPase RhoA, an inhibitor of axonal growth (Yu et al., 2011). Since the sciatic nerve is a heterogeneous tissue, the changes in miRNA expression may result from responses of both Schwann cells and neuronal cells to the nerve injury. This partially explains why some of the array data did not correlate well with the functions of the miRNA in neuronal cells. Only miR-21 was upregulated in both array data, which further confirmed the spatial specificity in miRNA expression (Wu et al., 2011).

## Conclusion

In the past decade, as one of the major discoveries in the history of molecular and cell biology, the post-transcriptional regulation of gene expression has become a major focus of research. There has been rapid progress in deciphering the mechanisms underlying miRNA pathway. The understanding of miRNA pathway in neuroscience is growing through intensive investigation of a variety of neurological events. Temporally and spatially specific miRNA expression has been identified in neurodevelopment and in neurological diseases (Martino et al., 2009; Smith et al., 2010; Martins et al., 2011). In axonal compartments, the miRNA machinery has been shown to be present and is functional upon application of siRNA to peripheral nerve fibers (Hengst et al., 2006; Murashov et al., 2007a). More importantly, ablating the miRNA processing negatively impacted peripheral nerve regeneration *in vivo* and regenerative axon growth *in vitro* (Wu et al., 2012).

The importance of local protein synthesis for nerve regeneration has been shown by an increase in local translation of proteins after peripheral nerve axotomy, and the observation that inhibiting this synthesis greatly reduces the reproduction of growth cones (Verma et al., 2005); however, the underlying mechanism responsible for the regulation of the local protein synthesis is largely unknown. Emerging evidence suggests miRNA could be one of the mechanisms that regulate axonal protein synthesis after peripheral nerve injury. However, we are only at the initial stage of understanding the role of miRNA in nerve injury and regeneration. Studies deciphering the functions mediated by miRNAs will have great significance in understanding basic cellular mechanisms, as well as inspiring miRNA-based therapeutics. As a mediator of gene silencing, miRNA has already shown therapeutic efficacy in animal models of neurological conditions. Further work will be required to elucidate how miRNA pathway contributes to peripheral nerve regeneration and how to use it as a tool to treat nerve injuries.

### Conflict of interest statement

The authors declare that the research was conducted in the absence of any commercial or financial relationships that could be construed as a potential conflict of interest.
